# Using the Remote Sensing and GIS Technology for Erosion Risk Mapping of Kartalkaya Dam Watershed in Kahramanmaras, Turkey

**DOI:** 10.3390/s8084851

**Published:** 2008-08-21

**Authors:** Alaaddin Yuksel, Recep Gundogan, Abdullah E. Akay

**Affiliations:** 1 Kahramanmaras Sutcu Imam University, Faculty of Forestry, Department of Forest Engineering, 46060, Kahramanmaras, Turkey; E-mail: ayuksel@ksu.edu.tr, akay@ksu.edu.tr; 2 Kahramanmaras Sutcu Imam University, Faculty of Agriculture, Department of Soil Science, 46060, Kahramanmaras, Turkey; E-mail: rgundogan@ksu.edu.tr

**Keywords:** RS, GIS, Kartalkaya Dam Watershed, CORINE

## Abstract

The soil erosion is the most serious environmental problem in watershed areas in Turkey. The main factors affecting the amount of soil erosion include vegetation cover, topography, soil, and climate. In order to describe the areas with high soil erosion risks and to develop adequate erosion prevention measures in the watersheds of dams, erosion risk maps should be generated considering these factors. Remote Sensing (RS) and Geographic Information System (GIS) technologies were used for erosion risk mapping in Kartalkaya Dam Watershed of Kahramanmaras, Turkey, based on the methodology implemented in COoRdination of INformation on the Environment (CORINE) model. ASTER imagery was used to generate a land use/cover classification in ERDAS Imagine. The digital maps of the other factors (topography, soil types, and climate) were generated in ArcGIS *v*9.2, and were then integrated as CORINE input files to produce erosion risk maps. The results indicate that 33.82%, 35.44%, and 30.74% of the study area were under low, moderate, and high actual erosion risks, respectively. The CORINE model integrated with RS and GIS technologies has great potential for producing accurate and inexpensive erosion risk maps in Turkey.

## 1. Introduction

Throughout the world water consumption is increasing more rapidly than the human population and has raised the socioeconomic and strategic importance of water resources. In order to continuously meet the growing per-capita demand for water, it reservoirs have to be built on rivers to deliver reliable supplies for public consumption [1]. Dams are one of the most important infrastructure investments in Turkey, providing essential services: drinking water, irrigation water, flood and torrent control, hydroelectric power, fisheries, wildlife, recreation, and other environmental benefits.

In Turkey, especially in the semi-arid and arid Mediterranean regions, soil erosion is one of the major threats to soil conservation and water resources. Soil erosion and sedimentation reduce the economic life of dams through the inflow and deposition of soil particles. In addition, sedimentation results in dramatic environmental impacts on water quality and aquatic habitat [2-3]. According to GDREC [4], over 345 million tones of sediment enters the rivers, lakes, dams, and seas per year in Turkey. Therefore, sustainable management and conservation of such expensive investments and their watersheds are crucial for the long-term quality of life and the national economy.

The amount of soil erosion is mainly affected by vegetation cover, topographic features, climatic variables, and soil characteristics. The human activities and large-scale developments alter the vegetation cover, impacting upon the soil erosion rate [5]. Topographic features such as ground slope, slope length, and shape most affect rill and interrill erosion [6]. The most important climatic variables are rainfall amount and precipitation intensity, which are called rainfall erosivity [6]. Besides, temperature is another important climatic variable since it affects the vegetative materials which are used in mulching to control erosion. Soil erodibility is mainly affected by aggregate stability, texture, depth, organic matter, and stoniness [7].

Assessing the soil erosion rate is essential for the development of adequate erosion prevention measures for sustainable management of land and water resources. Geographic Information System (GIS) technologies are valuable tools in developing environmental models through their advance features of data storage, management, analysis, and display [8]. The Remote Sensing (RS) technology has been used to provide the land use/cover information by using digital image processing techniques [9]. There have been many studies on modeling soil erosion by utilizing RS and GIS technologies [10-13].

The capabilities of these technologies even increase when they are integrated with empirical erosion prediction models [14]. While soil erosion models only calculate the amount of soil erosion based on the relationships between various erosion factors [15], RS and GIS integrated erosion prediction models do not only estimate soil loss but also provide the spatial distributions of the erosion [16]. Especially, generating accurate erosion risk maps in GIS environment is very important to locate the areas with high erosion risks [17] and to develop adequate erosion prevention techniques [18]. Sazbo *et al.*, 1998 [19] conducted a study where RS and GIS technologies were successfully used for land degradation and erosion mapping. Another study by Bojie *et al.* (1995) [20] also indicated that GIS analysis provide satisfactory results in developing erosion surveys and risk maps by using GIS data layers such as DEM, slope, aspect, and land use.

The most common empirical erosion prediction models, integrating with RS and GIS, are Revised Universal Soil Loss Equation (RUSLE), The Water Erosion Prediction Project (WEPP), and COoRdination of INformation on the Environment (CORINE), which can be used for erosion risk mapping. The RUSLE was developed to estimate the annual soil loss per unit area based on erosion factors including soil erodibility, topography, rainfall, and vegetation cover [21]. In the WEPP model, sediment yield and erosion rates were estimated for multiple time periods based on specific erosion factors [22].

To determine the erosion risks and qualities of the lands in the countries of European Union (EU), CORINE model was developed based on Universal Soil Loss Equation (USLE) [23] which is well-known methodology in soil erosion prediction studies. In CORINE model, actual soil erosion risk is determined by combining two parameters including potential soil erosion risk data and vegetation cover data. The potential soil erosion risk is calculated as a function of soil erodibility, erosivity, and topography. The vegetation cover data is very important parameter in erosion models since intensity of vegetation cover significantly affects erosion rates [6, 24-25]. Using high-resolution satellite imagery, image classification techniques have been used to generate accurate and reliable land use/cover data [9]. According to Zhu, 2001 [26] and Abrams, (2003) [27] accurate and low-cost land cover mapping can be provided by using the Advanced Spaceborne Thermal Emission and Reflection Radiometer (ASTER) images with high spatial (15 m × 15 m) and spectral (14 bands) resolution.

To accelerate future collaboration in watershed management between Turkey and EU, generating soil erosion risk maps based on the methodology used in CORINE model is crucial. This study uses RS and GIS technologies to develop soil erosion risk mapping for the Kartalkaya Dam Watershed in Kahramanmaras, Turkey, based on the CORINE model methodology. A supervised classification method was applied on ASTER imagery to classify land use/cover types. The input files for the other erosion factors (i.e. topography, soil types, and climate) were generated as GIS data layers and integrated into the CORINE model to produce erosion risk maps.

## 2. Material and Methods

### 2.1. Study Area

**Figure 1.**
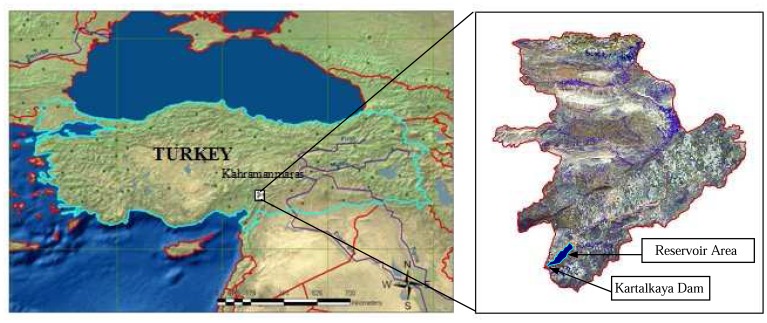
The location of the study area on the topographic map of Turkey and on the ASTER image.

The study area is located in the eastern Mediterranean region of Turkey, about 45 km southeast of the city of Kahramanmaras (Figure 1). The study area covers approximately 88100 ha of land with an elevation of 700 to 1850 m and slopes of 0 to 80 %. The land use/cover of the area contains agriculture, forest, rangeland, bare rock, water bodies, and residential areas. Average annual precipitation and temperature are 730 mm and 17.6 °C, respectively [28]. The highly erosive storms occur during fall and spring seasons. Kartalkaya, one of the most important dams in the eastern Mediterranean region of Turkey, supplies irrigation, domestic, and industrial water to the cities of Kahramanmaras and Gaziantep. The 56 m high dam stores about 2323 h m^3^ water in the approximately 11 km^2^ reservoir [29].

### 2.2. CORINE Erosion Model

**Figure 2.**
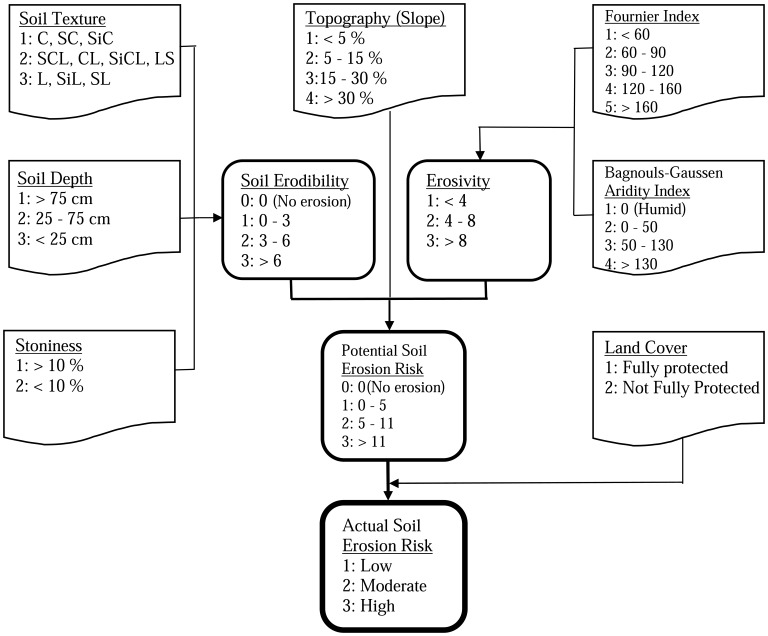
Flow diagram of CORINE method (Modified from [7]).

To estimate actual erosion risk in the CORINE model, the required database parameters are soil erodibility, erosivity, topography (slope), and land use/cover (vegetation cover) [7, 30]. The parameters are represented as four separate indices, which are then combined to evaluate erosion risk of the study area. Figure 2 indicates the logic behind the methodology used in CORINE model.

#### 2.2.1. Soil Erodibility

In CORINE methodology, soil erodibility is calculated by considering soil texture, soil depth, and stoniness. In terms of soil texture, silt, very fine sand, and clay soils tend to be less erodible than sand, sandy loam, and loamy soils [7]. The existence of stones over the soil surface may reduce erosion by protecting soil from rain splash. However, after surface runoff is initiated, existence of stones may cause adverse effects by encouraging rill erosion through water turbulences. Increasing the soil depth results in a higher water holding capacity, which may prevent overland flow by absorbing larger amounts of rainfall [7].

In the CORINE model, soil texture is classified into three classes including (1) slightly erodible, (2) moderately erodible, and (3) highly erodible according to the USDA textural classification [30] (Figure 2). Similarly, the soil depth is also classified as (1) slightly erodible, (2) moderately erodible, and (3) highly erodible soils, by considering the depth from the soil surface to the base of the soil profile. By considering the percentage surface cover of stones, the stoniness is classified as (1) fully protected and (2) not fully protected soils.

Finally, the soil erodibility index can be calculated as a function of soil texture, soil depth, and stoniness [7, 32-34]:

(1) Soil Erodibility Index=Texture Class×Depth Class×Stoniness Class

In this study, first, GIS data layer for each parameter (i.e. each soil texture, soil depth, and stoniness) was generated based on using 1:25000 scaled soil map [35] and refined by comparing with soil samples collected from 87 plots of known locations (i.e. using GPS) in the study area. In order to validate the overall erosion risk map, erosion survey was also performed for each plot by implementing qualitative assessment method [36]. The soil properties such as the aggregate stability, permeability, organic matter, carbonate, and texture were analyzed in the Soil Laboratory [36]. Then, GIS data of each layer has been recorded according to the CORINE methodology indicated in Figure 2.

The soil erodibility map was produced by applying “Raster Calculator” tool in “Spatial Analyst” extension of ArcGIS *v*9.2.[37] Finally, the soil erodibility index was calculated (Equ. 1) and reclassified into three classes including (1) low, (2) moderate, and (3) high erosion. When there is no soil cover (e.g. bare rock, urban land, and water), the value of the index is equal to 0, which indicates that there is no erosion in the area [7, 30, 32].

#### 2.2.2. Erosivity

Erosivity, which is defined as detachment and transportation of soil due to raindrop impact and runoff, primarily depends on the intensity and the amount of rainfall [6]. In CORINE model, erosivity is calculated by combining two climatic indexes including the Fournier index and Bagnouls-Gaussen aridity index (BGI).

Fournier index was developed specifically to measure erosivity at a regional scale [38]. The modified Fournier index (MFI) is computed depending on total precipitation in a month (*P_i_*) and total mean annual precipitation (*P_a_*) as follows [39]:

(2) $$\text{MFI}=\sum_{i=1}^{12}\frac{{P_{i}}^{2}}{P_{a}}$$

In CORINE model, the MFI is classified into five classes including (1) very low, (2) low, (3) moderate, (4) high, and (5) very high. On the other hand, the MFI could not consider the moisture stress which may increase the soil erosion due to reduction of vegetation cover. Thus, the BGI is employed as the second climatic index to consider moisture stress in terms of the ratio of the temperature and precipitation. The BGI is defined as follows [7, 39]:

(3) $$\text{BGI}=\sum_{i=1}^{12}\left(2t_{i}-P_{i}\right)k_{i}$$

where *t_i_* is the mean temperature for the month, *P_i_* is the total precipitation for month, and *k_i_* is the proportion of the month during which *2t_i_*-*P_i_*> *0*. The BGI is classified into four classes; (1) humid, (2) moist, (3) dry, and (4) very dry. Finally, erosivity index is determined by combining these two climatic indices as follows:

(4) Erosivity Index=Variability Class×Aridity Class

In this study, necessary input data of precipitation and temperature were obtained from the nearest Meteorology Observation Station for the period of 1985-2007 [28]. Then, erosivity index was reclassified into three classes; (1) low, (2) moderate, and (3) high.

#### 2.2.3. Topography (Slope)

One of the key factors in soil loss is topography, especially, when the ground slope exceeds a critical angle [7]. The slope data layer can be accurately and quickly generated in GIS environment from topographic maps, digital terrain models, satellite imagery, or other sources. In the CORINE model, the topographic factor is defined in terms of the average regional slope. In this study, the digital topographic maps with the scale of 1:25000 from the General Command of Mapping-Turkey, were used to generate a Digital Elevation Model (DEM) of the study area. Then, the slope data layer was derived from the DEM data and classified into four classes according to the CORINE methodology. The slope layer was derived from the DEM and classified into four classes; 1- very gentle to flat (<5%), 2- gentle (5-15%), 3- step (15-30%), and 4- very steep (>30%).

#### 2.2.4. Vegetation Cover

Vegetation significantly reduces the erosion rate by intercepting raindrops [6]. The vegetation cover results in better water-holding capacity, reduces runoff, and improves infiltration. Besides, the vegetation cover type can be altered into ideal forms to reduce erosion. Thus, reliable land use/cover data is crucial in soil erosion models [21, 32-33, 40-41] The RS technology provides accurate and inexpensive land use/cover data layer by using digital image processing techniques [9]. In CORINE model, vegetation cover layer is divided into two classes: 1) fully protected (forest, permanent pasture, and dense scrub) and 2) not fully protected (cultivated or bare land) [7].

For this study, a vegetation cover map was generated using a supervised classification method applied to cloud-free on ASTER imagery acquired on the 16^th^ of August, 2005. First, the study area was clipped out from the ASTER image by using “Subset” function in ERDAS 8.5 [42]. The image was georeferenced based on 1:25 000 scale topographic maps and then re-projected into the UTM projection zone 37 and ED 50 datum. The overall RMS error was less than 0.5 pixels using a second order polynomial model. To increase the accuracy of classification process, the ASTER imagery was converted into the top-of-atmosphere (TOA) reflectance by using the procedure explained by Yuksel *et al.* [9] in detail. Then, the low-pass filtering technique (7×7) was applied to reduce spatial frequency of data variability [43].

In land cover classification, a Supervised Classification method was performed by collecting 87 training points from TOA reflectance data. The land use/cover classes of these training points were determined based on the general knowledge obtained from infrared air photos, topographic maps, and field visits. Then, the parallelepiped non-parametric and minimum distance parametric rule was used to perform supervised classification method. The classified image was recoded into seven main classes including forest (1), irrigated crops (2), stubble (3), fallow (4), rangeland (5), water body (6), and bare land and residential (7). After recoding process, accuracy assessment was performed based on stratified random sampling method where 256 points were automatically selected from referenced topographic map and air photos. Then, user's accuracy and producer's accuracy of each class was used to compute overall accuracy and kappa values. Finally, the classified vegetation cover image was reclassified into two classes (i.e. fully protected and not fully protected) as defined by the CORINE model.

#### 2.2.5. Actual Erosion Risk

In this stage, firstly, soil erodibility, erosivity, and topography layers were overlapped by applying the “Raster Calculator” tool in the “Spatial Analyst” extension of ArcGIS *v*9.2 [37] in order to calculate the potential soil erosion risk of the study area. The potential soil erosion risk is formulated as follows:

(5) Potential Soil Erosion Risk Index=Soil Erodibility Index×Erosivity Index×Slope index

Then, the vegetation cover layer is combined with the potential soil erosion risk layer in ArcGIS *v*9.2 [37] to generate actual soil erosion risk map (Figure 2). Finally, actual soil erosion risk map is classified in to three classes; (1) low, (2) moderate, and (3) high.

## 3. Results and Discussion

**Figure 3.**
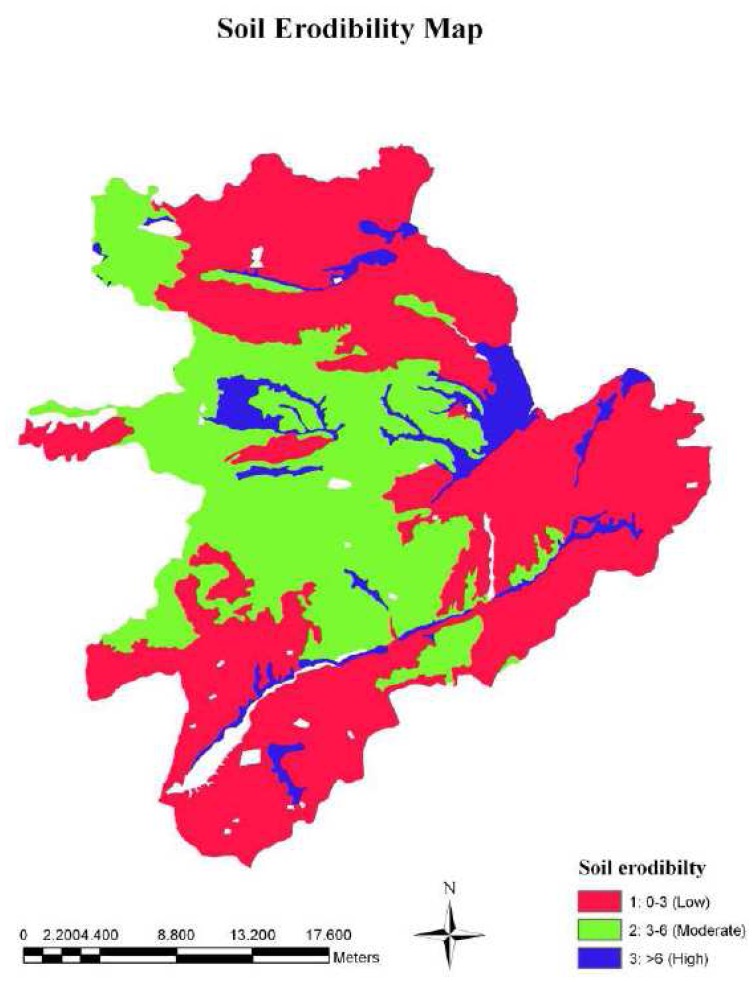
The soil erodibility layer of the study area.

The results indicated that the soil texture classes in the study area were clay (58%), sandy clay loam (35%), and loam and silty loam (7%). Therefore, the soils of the study area have the ability to resist soil erosion since clay soil and sandy clay loam soil are less susceptible to erosion [6]. In the study area, the soils were very shallow (17%), shallow (23%), or moderately deep (16%), which resulted in high erosion rate due to lower water holding capacity and higher overland flow [7]. The results also indicated that 55% of the study area had less than 10% stoniness, while rest of the area has more than 10% stoniness. Then, the soil erodibility map of the study area was generated by overlapping soil texture, depth, and stoniness layers (Figure 3). About 48% of the study area was covered by moderately erodible soils, while 38% and 14% was covered by low and highly erodible soil, respectively.

The MFI of the study area was calculated as 110, which was classified as moderate (3) according to the CORINE model. The BGA index was found to be 140 for the study area and classified as very dry (4). Then, the MFI and the BGA indices were combined to generate the erosivity layer. The results indicated that erosivity index was determined as high (12) for the study area.

**Figure 4.**
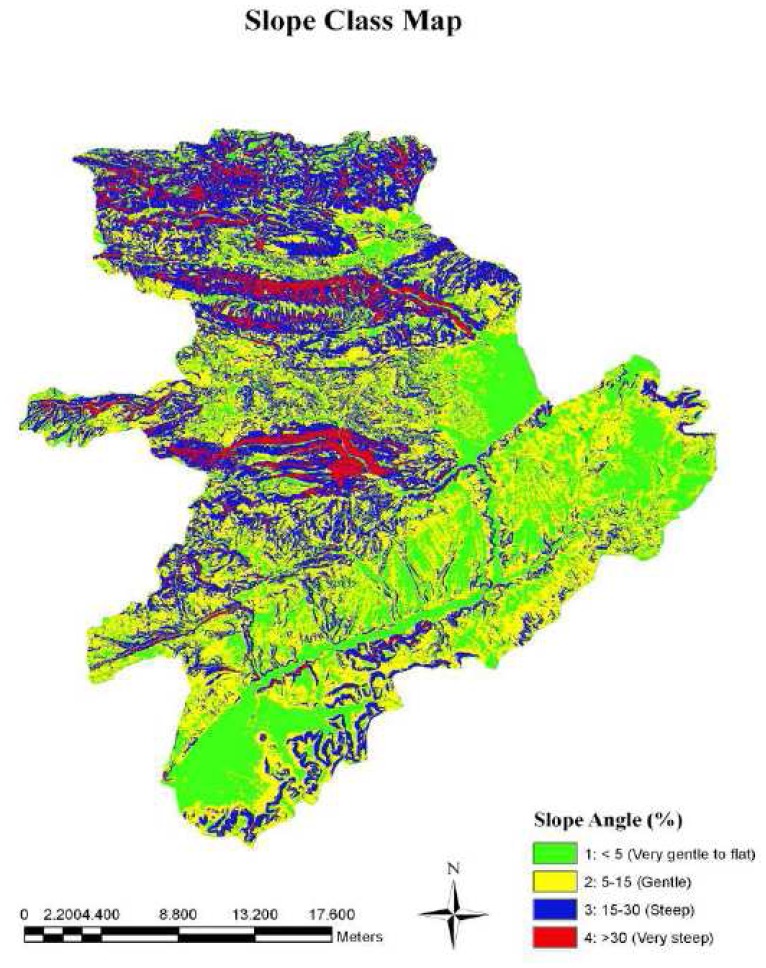
The slope layer of the study area.

The slope data layer was derived from DEM of the study area and classified according to CORINE model (Figure 4). The results showed that about 38% of the study area had the slope of more than 15%, ranging from steep to very steep terrain, which may significantly increases the soil erosion due to runoff [6]. The rest of the study area lies on terrain with less than 15% slope, ranging from very gentle to gentle.

**Figure 5.**
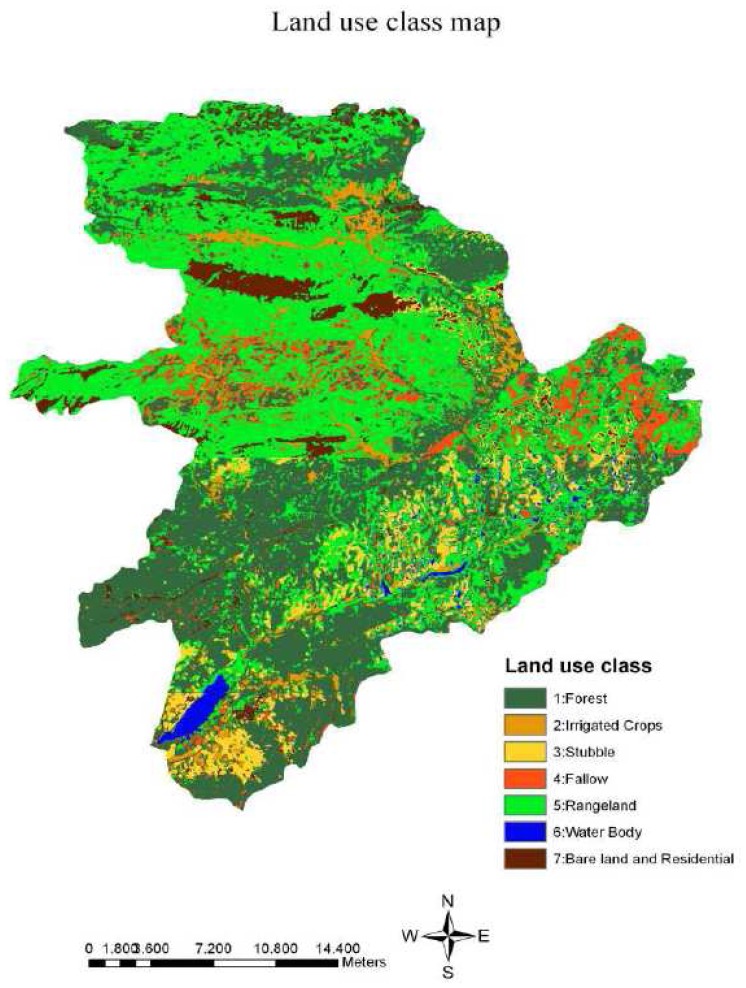
Land use/cover classes after supervised classification of the study area.

To generate vegetation cover layer, the supervised classification was applied considering seven main land use/cover classes (Figure 5). The accuracy assessment of the classified image indicated that classification process provided overall accuracy and kappa values of 78.91% and 0.73, respectively (Table 1). The classification provided satisfactory results in terms of distinguishing water body, bare land and residential, stubble, and rangeland; however, accuracy for fallow, forest, and irrigated crops was relatively low due to large variation of spectral signatures. The highest producer's accuracy was reached in classification of water bodies (100%), followed by forest, stubble, and range land.

The highest user's accuracy was reached in classification of water body and irrigated crops (100%), followed by rangeland and stubble. The lowest producers and users accuracy was reached in classification of fallow by 46.15% and 63.16, respectively. It was assumed that the accuracy of the fallow was low because the reflection values received from fallows and forest were close, especially in the areas where sparse forests exist. The results also indicated that supervised classification overestimated forest and stubble, while it underestimated irrigated crops, fallow, rangeland, and bare land and residential. In the lands with high moisture content, irrigated crops were classified as forest. The stubble reflected high reflection values similar to bare land and residential.

The vegetation cover layer is reclassified into two vegetation indices including fully protected and not fully protected based on CORINE method. In the study area, forest was classified as fully protected, while irrigated crops, stubble, fallow, and rangeland were classified as not fully protected.

**Figure 6.**
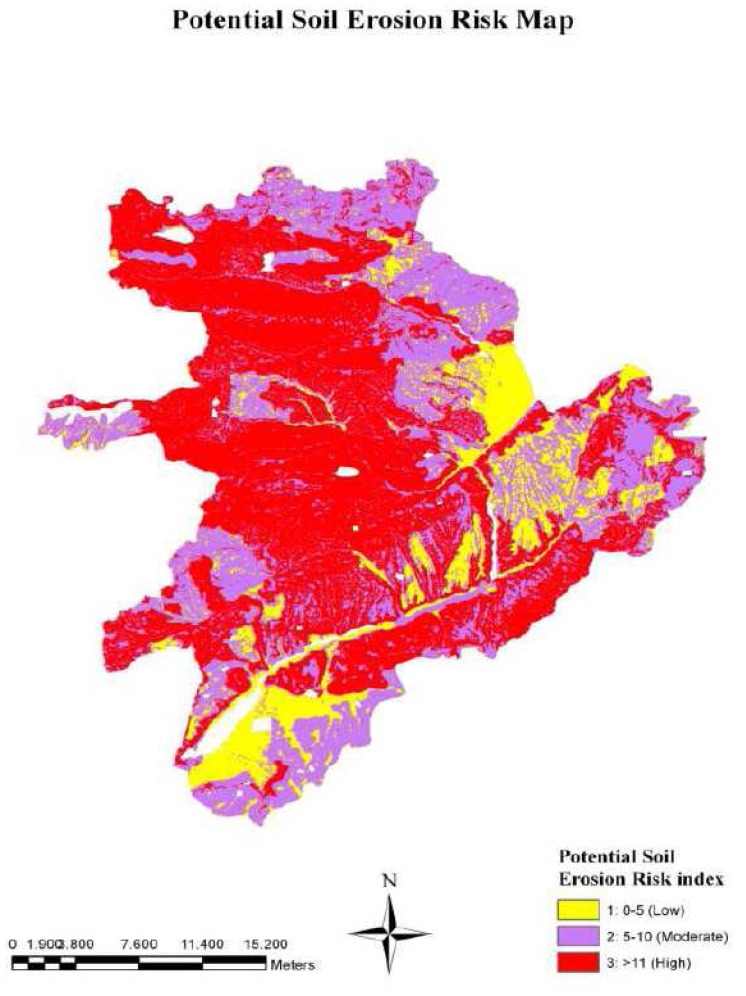
The potential soil erosion risk layer of the study area.

Finally, the potential erosion risk map was generated by overlapping soil erodibility, erosivity, and slope layers (Figure 6). Then, the land cover map and the potential erosion risk map were combined to produce the actual soil erosion risk map.

The results presented in Table 2 showed that about 18% of the study area was classified as low potential erosion risk, while rest of the area was under moderate to high potential erosion risk. In terms of actual soil erosion risk, the study area has 35% moderate, 34% low, and 31% high erosion risk levels. The results also indicated that the areas with moderate and high erosion risk located in the north and northwest of the study area, while the areas with low erosion risk located in the southwest and east of the study area.

**Figure 7.**
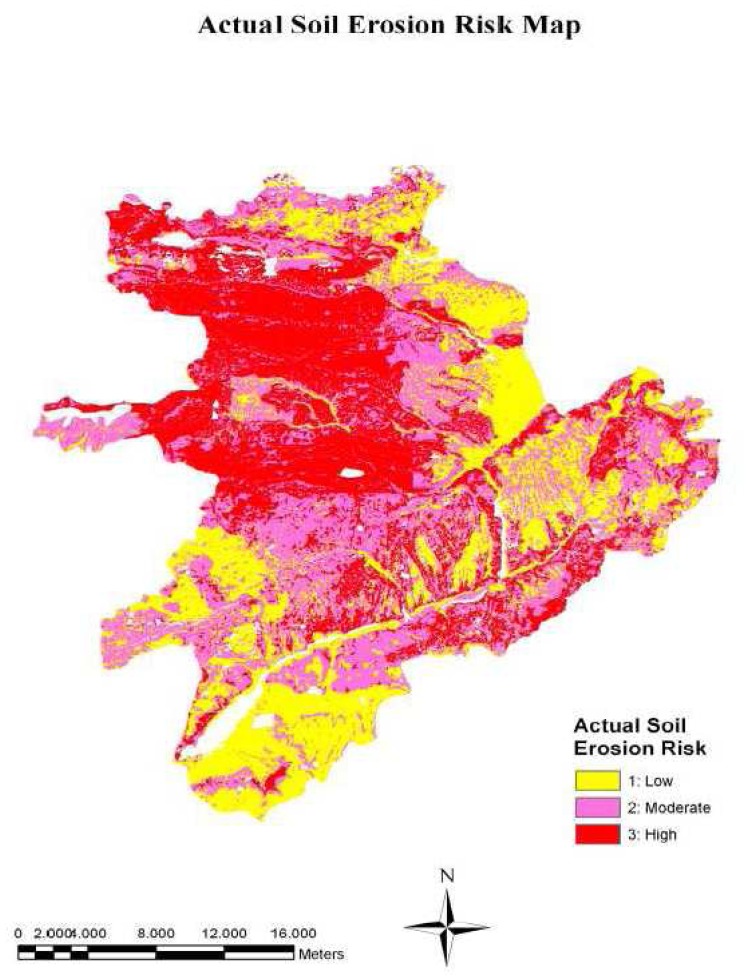
The actual soil erosion risk layer of the study area.

The difference between the areas of potential and actual erosion risk indicates the effects of vegetation cover on soil erosion. The areas classified as high erosion risk in the potential erosion risk map were reduced from 47.81% to 30.74% in actual soil erosion risk map, after overlapping the vegetation layer. This proved that the areas subject to high erosion risk are mostly covered by forest vegetation and rangeland as indicated in Figure 7. On the other hand, the total areas classified as low and moderate erosion risk in the potential erosion risk map were increased from 52.19% to 69.26% in actual soil erosion risk map, due to inappropriate agricultural practices, over grazing, and deforestation.

The validation process based on soil survey from 87 plots indicated that the accuracy of actual erosion risk map was approximately 86%. Therefore, there was a good agreement between actual soil erosion risk map and soil survey data.

## 4. Conclusion

This study indicated that using RS and GIS technologies for erosion risk mapping, based on the methodology implemented in CORINE model, resulted in effective and accurate assessment of soil erosion in considerable short time and low cost for large watersheds. Even though this method requires advanced RS and GIS technologies and sophisticated computer involvement, the users with basic computer skills can implement it without any assistants from the computer specialists. The model did not intended to estimate the amount of soil loss and sediment yield but to provide erosion risk map for the analysis of planning and environmental protection. Besides, the model can provide the decisionmakers with the areas with erosion risk so that they can develop soil and water conservation plans in general and generate detailed erosion studies for the areas of high erosion risk.

## Figures and Tables

**Table 1.** The results from the accuracy assessment process.

Class Names	Reference Data								

	Forest	Irrigated Crops	Stubble	Fallow	Range Land	Water Body	Bare Land/Residential	Total	User's Accuracy

Forest	75	10	1	10	6		1	103	72.82
Irrigated Crops		15						15	100.00
Stubble	2		35				5	42	83.33
Fallow	3	1		12	3			19	63.16
Range Land	2			3	39		1	45	86.67
Water Body						5		5	100.00
Bare Land/Residential	1		3	1	1		20	26	76.92

Total	83	26	39	26	49	5	27	256	

Producer's Accuracy	90.36	57.69	89.74	46.15	79.59	100.00	74.07		

**Table 2.** The area of the potential and actual soil erosion risk indices for the study area.

Index Values	Potential Erosion Risk		Actual Soil Erosion Risk	

	Area (ha)	Percentage	Area (ha)	Percentage
1	15870	18.01	29797	34
2	30112	34.18	31221	35
3	42120	47.81	27084	31
Total	88102	100.00	88102	100
